# Cotton *GalT1* Encoding a Putative Glycosyltransferase Is Involved in Regulation of Cell Wall Pectin Biosynthesis during Plant Development

**DOI:** 10.1371/journal.pone.0059115

**Published:** 2013-03-18

**Authors:** Li-Xia Qin, Yue Rao, Long Li, Jun-Feng Huang, Wen-Liang Xu, Xue-Bao Li

**Affiliations:** Hubei Key Laboratory of Genetic Regulation and Integrative Biology, College of Life Sciences, Central China Normal University, Wuhan, China; East Carolina University, United States of America

## Abstract

Arabinogalactan proteins (AGPs), are a group of highly glycosylated proteins that are found throughout the plant kingdom. To date, glycosyltransferases that glycosylate AGP backbone have remained largely unknown. In this study, a gene (*GhGalT1*) encoding a putative β-1,3-galactosyltransferase (GalT) was identified in cotton. GhGalT1, belonging to CAZy GT31 family, is the type II membrane protein that contains an N-terminal transmembrane domain and a C-terminal galactosyltransferase functional domain. A subcellular localization assay demonstrated that GhGalT1 was localized in the Golgi apparatus. RT-PCR analysis revealed that *GhGalT1* was expressed at relatively high levels in hypocotyls, roots, fibers and ovules. Overexpression of *GhGalT1* in *Arabidopsis* promoted plant growth and metabolism. The transgenic seedlings had much longer primary roots, higher chlorophyll content, higher photosynthetic efficiency, the increased biomass, and the enhanced tolerance to exogenous D-arabinose and D-galactose. In addition, gas chromatography (GC) analysis of monosaccharide composition of cell wall fractions showed that pectin was changed in the transgenic plants, compared with that of wild type. Three genes (*GAUT8*, *GAUT9* and *xgd1*) involved in pectin biosynthesis were dramatically up-regulated in the transgenic lines. These data suggested that GhGalT1 may be involved in regulation of pectin biosynthesis required for plant development.

## Introduction

Plant cell wall is a complex composite of cellulose, hemicelluloses and pectins, and plays a critical role in growth and morphogenesis [1]. In addition to these polysaccharides, the cell wall contains abundant hydroxyproline-rich glycoproteins (HRGPs). HRGPs represent one superfamily of plant cell wall proteins that can be divided into three subclasses, i.e. highly glycosylated arabinogalactan-proteins (AGPs), moderately glycosylated extensins (EXTs), and lightly glycosylated proline-rich proteins (PRPs) [2], [3]. These proteins have their proline residues which are hydroxylated to hydroxyproline by prolyl 4-hydroxylases (P4Hs) and then are extensively O-glycosylated by glycosyltransferases in the Golgi apparatus or endoplasmic reticulum (ER) [4].

AGPs are defined by the presence of arabinogalactan (AG) polysaccharides and reside mainly at plasma membrane-cell wall interface and in plant exudates [5]. The very high ratio of carbohydrate to protein in AGPs (i.e., 90%–10% respectively) suggests that the glycan side chains of AGPs are important in their functioning as well as their interactions with other proteins [6]. These AG polysaccharides are added by O-glycosylation, such as galactosylation in serine residues and arabinosylation/or arabinogalactosylation in hydroxyproline residues [7].

In *Arabidopsis* and rice, 85 and 69 AGPs have been identified, respectively [5], [8]. AGPs have been implicated in various aspects of plant growth and development, including cell proliferation, cell expansion, programmed cell death, pollen tube growth, xylem differentiation, somatic embryogenesis, zygotic division, and embryo development [8]–[10]. However, only a few AGPs have been functionally elucidated by analyzing mutants. For example, *Arabidopsis rat1* (resistant to *Agrobacterium* transfomation) mutant with a T-DNA insertion in the promoter region of AtAGP17 is defective in binding of *Agrobacterium* to its roots in water solution [11]. An *AtAGP18* RNAi mutant exhibits defective ovule development as functional megaspores fail to enlarge and divide [12]. The *atagp19* mutant displays a lot of abnormalities (including smaller, rounder and lighter-green rosette leaves, delayed growth, shorter inflorescence stems and fewer siliques) indicating AtAGP19 functions in various aspects of plant growth and development [9]. The mutant of *AtFLA4*, sos5 (salt overly sensitive5), demonstrates abnormal cell expansion, thinner cell walls and increased sensitivity to salt [13]. Recently, *Atfla1* is found to be deficient in shoot regeneration [14].

Currently, most studies are focused on AGP protein backbone comprising Hyp/Pro, Ala, Ser, and Thr residues. Little is known about the roles of AG side chains and none specific glycosyltransferase involved in side chain synthesis has been characterized. It has been suggested that the carbohydrate moieties of AGPs are important for AGP function. In *Nicotiana alata*, fully glycosylated transmitting tissue-specific (TTS) protein was involved in promoting pollen tube growth and impacting pollen tube guidance, but did not the deglycosylated form of TTS [15]. The reduction of fucose in AGP side chain in the roots of *Arabidopsis* mutant *mur1* caused a 33%–50% decrease in root cell elongation [16]. Addition of a single AG glycosylation site to human growth hormone significantly increased its secretion from tobacco suspension cells [17].

Structural analysis of the AG polysaccharide domain and functional characterization of transferase specificity have shown that tobacco uses 15 transferases in AG synthesis [18]. Though AGPs are found ubiquitously throughout the entire plant kingdom and have been isolated from a wide range of plant organs and cell types, there is an embarrassing lack of knowledge on the enzymology of AGP biosynthesis during the past decade [3]. In recent years, some progress has been made in this field. For example, 20 putative *Arabidopsis* β-(1,3)GalTs were identified by searching *Arabidopsis* homologs to mammalian β-(1,3)GalTs, one of which is involved in the biosynthesis of Lewis A structure (a trisaccharide [Fucα1-4(Galβ1-3)GlcNAc-R]) -bound *N*-linked oligosaccharide [19], [20]. Biochemical analysis indicated that two α-(1,2) fucosyltransferases, AtFUT4 and AtFUT6, which belong to glycosyltransferase 37 family (GT37), could specifically add fucose to the end of AG side chain on AGP [21]. Additionally, an *in vitro* assay system of Hyp-O-galactosyltransferase (HGT) activity has been established to detect and localize Hyp:GalT activity in the endoplasmic reticulum (ER) of *Arabidopsis* [22]. Subsequently, GalT proteins have been proven to participate in the initial steps of AGP glycosylation in both tobacco and *Arabidopsis* by analyzing GalT activities *in vitro* [23]. Particularly, At1g53290 and At3g14960, which encode plant specific β-GalTs, may be involved in the synthesis of AGPs [6], [24].

Cotton (*Gossypium hirsutum*) is an important fiber crop in the world. In our previous study, 21 genes encoding AGPs were isolated from cotton cDNA libraries, and 8 of them were predominantly expressed in 10 DPA fibers [25]–[27]. Recently, Yuan et al (2011) generated 10,979 ESTs from a normalized fiber cDNA library of *G. barbadense*. These ESTs were assembled into 5852 unigenes, 282 of which were identified as cell wall biosynthesis related. Very interestingly, the most abundant cell wall gene category was AGP genes [28]. The above results indicate AGPs may be important players during fiber development. In addition, immunofluorescence assays by JIM13, JIM14, LM2, and CCRC-M7 antibodies recognizing glycan moiety epitopes on AGPs showed different labeling patterns of AGPs in developing fiber cells, suggesting polysaccharide side chain of AGPs might be critical participants in the early stages of initiation and elongation of cotton fibers [29], [30]. Moreover, comparative proteomics indicated that biosynthsis of pectic precursors is important for cotton fiber elongation [31]. However, no glycosyltransferase involved in biosynthesis of AGP glycan side chains in cotton has been characterized. Here, we report the characterization of a cotton gene encoding an AGP-glycosyltransferase (designated GhGalT1). The transgenic *Arabidopsis* seedlings overexpressing *GhGalT1* displayed much longer primary roots, higher chlorophyll content, higher photosynthetic activity and increased biomass. Further study revealed that pectin content of cell walls was changed, and three genes (*GAUT8*, *GAUT9* and *xgd1*) involved in pectin biosynthesis were dramatically up-regulated in the transgenic plants.

## Materials and Methods

### Cotton Material and Growth Conditions

Cotton (*Gossypium hirsutum*, cv. Coker 312) seeds were surface-sterilized with 70% (v/v) ethanol for 1 min and 10% (v/v) H_2_O_2_ for 2 h, and then washed with sterile water. The sterilized seeds were germinated on 1/2 Murashige-Skoog (MS) medium under a 16 h light/8 h dark cycle for 6 days at 27°C. Roots, cotyledons and hypocotyls were cut from sterile seedlings. Other tissues for RNA extraction were derived from cotton plants grown in the soil.

### DNA and Protein Sequence Analysis

Unless otherwise stated, nucleotide and amino acid sequences were analyzed using DNAstar (DNAstar Inc). Identification of protein domains and significant sites was performed with Motifscan (http://myhits.isb-sib.ch/cgi bin/motif_scan). Signal P (www.cbs.dtu.dk/services/SignalP/) was used to determine the N-terminal signal sequence.

### Phylogenetic Analysis

A phylogenetic analysis was performed in order to investigate the evolutionary relationships among the GhGalT1 and other related galactosyltransferases (GalTs). GhGalT1 and other plant galactosyltransferase (GalT) sequences were aligned with the Clustal X program, and then the Neighbor-joining tree was constructed in MEGA 5.0 from 1000 bootstrap replicates [32].

### Subcellular Localization of GhGalT1 Protein

The coding sequence of *GhGalT1* gene (without the stop codon) was cloned into the pBI121-eGFP vector (without *GUS* gene) at a position upstream of the *eGFP* gene at *Xbal* I site. The *GhGalT1:eGFP* construct was introduced into *Agrobacterium tumefaciens* GV3101 strain. *Agrobacterium* containing the *GhGalT1:eGFP* or Golgi apparatus marker GONST1:YFP [33] constructs were grown to saturation in Luria-Bertani (LB) medium. Cultures were centrifuged and resuspended in 10 mM MgCl_2_, 10 mM MES, and 150 mM acetosyringone and kept at room temperature for 2 h. The cultures were then diluted to 1 OD_600_ unit and coinfiltrated into the abaxial side of a young tobacco (*Nicotiana tabacum*) leaf epidermis (four-week-old seedlings grown at 22°C) using a 2 ml syringe without the needle. Transformed leaves were analyzed 48–72 h after infection of the lower epidermis [34]. Subsequently, fluorescence microscopy was performed on a SP5 Meta confocal laser microscope (Leica, Germany). SP5 software (Leica, Germany) was employed to record and process the digital images taken. At least three independently transformed leaves were analyzed. Primers used in *GhGalT1:eGFP* vector construction as follows: GhGalT1 P1∶5′-GGGTCTAGAATGCCATCCTCTCCCAAG-3′; GhGalT1 P2∶5′-GGGTCTAGAATCATCGTCCGATGGCAA-3′.

### Phenotypic Analysis of Transgenic *Arabidopsis* Seedlings

The coding sequence of *GhGalT1* gene, amplified from its cDNA by PCR with the proofreading *pfu* DNA polymerase, was cloned into pBI121 vector at *Bam*H I/*Sac* I sites to replace the *GUS* gene. Primers used as follows: GhGalT1 P1∶5′-GGGTCTAGAATGCCATCCTCTCCCAAG-3′; GhGalT1 P2∶5′-CTTGAGCTCATTCTGACACCTTTCCATG-3′. The construct was then transferred into *Arabidopsis* by the floral dip method. Positive transformants were selected on MS medium with 50 mg/L kanamycin and grew until maturation and seed set. The *GhGalT1*-overexpression transgenic lines were named as *GhGalT1oe.* Homozygous lines of T3 and T4 generations were used for phenotypic analysis.

Total RNAs were extracted from one-week-old seedlings of *GhGalT1oe* and wild type, 21-day-old stems of At1g53290 T-DNA insertion mutant (SALK 015338) and 42-day-old flowers of At3g14960 T-DNA insertion mutant (SALK 043252), as At1g53290 (AtbGalT1) and At3g14960 (AtbGalT2) share high sequence similarity with GhGalT1. Semi-quantitative RT-PCR analysis was performed as described as above.

Seeds of wild type, independent lines of *GhGalT1oe* transgenic plants, and single mutants of SALK 015338 and SALK 043252 were respectively germinated on 1/2 MS medium supplemented with or without different concentrations of sucrose, D-galactose (Gal) and D-arabinose (Ara). The seeds were incubated at 4°C for 3 days before being placed at 22°C under light conditions (16 h light/8 h dark cycle). For culturing in the vertical position, all above type seeds were placed in rows on the plates. Changes in root growth of nine-day-old seedlings were subsequently monitored and recorded everyday during growth of the plants.

Nine-day-old *Arabidopsis* seedlings of *GhGalT1oe*, wild type and T-DNA insertion mutant were transferred and cultured at 4°C for 3 days, and then the chlorophyll content and photosynthetic efficiency in leaves of both transgenic and wild type plants were determined, respectively. In brief, chlorophyll in 0.1 g leaves was extracted with 10 mL of 80% acetone, and chlorophyll content was assayed by measuring absorbance at 645 and 663 nm with a spectrophotometer [27]. The assays were repeated three times along with three independent repetitions of the biological experiments.

### Hypocotyl Measurements

The seeds were incubated at 4°C for 3 days before being placed at 22°C under fully dark conditions. For culturing in the vertical position, all above seeds were placed in rows on 1/2 MS medium supplemented with 1% sucrose or without any sugar. Changes in hypocotyl growth of nine-day-old seedlings were subsequently monitored and recorded everyday during the growth of the plants. The assays were repeated three times along with three independent repetitions of the biological experiments.

### Extraction, Separation and Analysis of Cell Wall Polymer Fractions

*Arabidopsis* plants grew in pots for approximately 20 days and placed in the dark for 3 days. Rosette leaves harvested from these plants were lyophilized and ground into a fine powder, followed by washing three times with 70% ethanol, three times with 1∶1 methanol-chloroform, and two times with acetone to obtain alcohol insoluble residue (AIR). Aliquots of 200 mg AIR were sequentially extracted with 50 mM CDTA pH 6.5, 50 mM Na_2_CO_3_ (with 10 mM NaBH_4_), 1 M KOH (with 10 mM NaBH_4_) and 4 M KOH (with 10 mM NaBH_4_). All fractions were filtered by glass microfiber filters (GE Healthcare UK Limited), dialyzed extensively in cellulose ester dialysis membranes against water, and subsequently lyophilized.

The non-cellulosic monosaccharide composition of the wall matrix polysaccharides was obtained by treating AIR with trifluoroacetic acid and subsequent derivatization of the solubilized monosaccharides into their corresponding alditol acetates followed by quantification by gas chromatography (GC) [35], [36].

### RT-PCR Analysis

Total RNA was extracted from different cotton tissues. The expression of *GhGalT1* gene in cotton tissues was analyzed by quantitative RT-PCR using the fluorescent intercalating dye SYBR-Green in a detection system (Opticon2; MJ Research) as described previously [37]. The cotton polyubiquitin gene (*GhUBI1*, access number in GenBank: EU604080) was used as an internal control in Real Time quantitative RT-PCR reactions. The primer sequences were as follows: *GhGalT1* forward 5′-GATGATGATATATATTTGAG-3′ and *GhGalT1* reverse 5′-CTCACTCAGACTTACCTTGGTTGG-3′; *GhUBI1* forward 5′-CTGAATCTTCGCTTTCACGTTATC-3′ and *GhUBI1* reverse 5′-GGGATGCAAATCTTCGTGAAAAC-3′.

For analyzing cell wall-associated gene expression in transgenic plants, total RNA was isolated from the ten-day-old *Arabidopsis* seedlings of wild type, GhGalT1oe, and SALK 015338 and SALK 043252 mutants with three biological replicates using the RNeasy Mini Protocol (Qiagen). Semi-quantitative and real-time quantitative RT-PCR analyses were performed with gene-specific primers for cell wall-associated genes using *ACTIN2* as a normalization control. The expression levels of genes involved in the biosynthesis of cellulose in primary cell wall (*CesA1*, *CesA3* and *CesA6*), cellulose in secondary cell wall (*CesA4*, *CesA7* and *CesA8*), xyloglucan (*XXT1*, *XXT2* and *XXT5*), xylan (*FRA8* and *IRX12*), pectin (*GAUT1*, *GAUT8*, *GAUT9*, *Qua2* and *xgd1*) and lignin (*4CL* and *CCoMoAT1*) were examined [38].

## Results

### Isolation and Characterization of *GhGalT1* Gene

**Figure 1:**
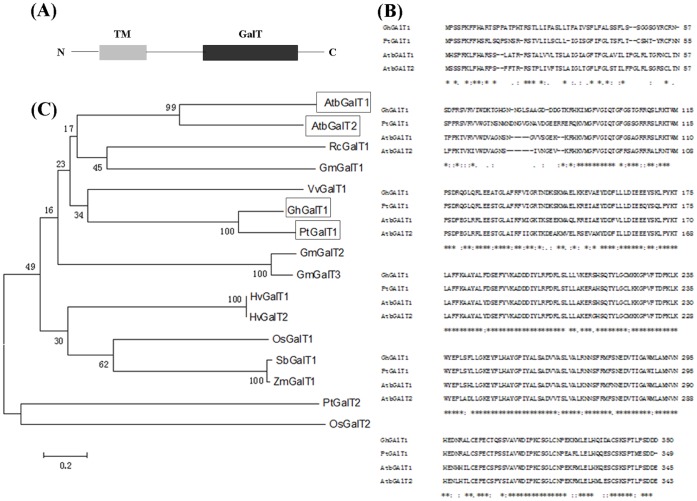
Characterisation of GhGalT1. (**A**) Structure of GhGalT1 protein. N: N-terminal; TM: transmembrane domain, GalT: the conservative galactosyltransferase functional domain; C: C-terminal. (**B**) Comparison of the predicted amino acid sequence of GhGalT1 with some known GalT proteins. The alignment was generated with the CLUSTAL W program. The same nucleotides are highlighted by asterisk, conserved and similar residues are indicated by colon or dot mark. (**C**) Phylogenetic relationships between GhGalT1 and other GalTs in plants. A neighbour-joining tree was generated with MEGA5 from 1000 bootstrap replicates. The accession numbers of these known proteins in GenBank are: *Gossypium hirsutum* GhGalT1 (JX448620); *Populus trichocarpa* PtGalT1 (XP_002330737) and PtGalT2 (XP_002317466); *Arabidopsis thaliana* AtbGalT1 (At1g53290) and AtbGalT2 (At3g14960); *Zea mays* ZmGalT1 (NP_001152267); *Vitis vinifera* VvGalT1 (XP_002283081); *Ricinus communis* RcGalT1 (XP_002524276); *Glycine max* GmGalT1 (XP_003543216), GmGalT2 (XP_003556804) and GmGalT3(XP_003527208); *Oryza sativa* OsGalT1 (Os06g0156900) and OsGalT2(CAD44839.); *Hordeum vulgare* HvGalT1 (BAJ94890) and HvGalT2(ABL11234); *Sorghum bicolor* SbGalT1 (XP_002436508).

Since Qu et al. (2008) proposed that members of CAZy glycosyltransferase31 (GT31) family may be involved in the synthesis of the AG polysaccharide on AGPs. Subsequent assay *in vitro* of GalT activity also suggested that members of GT31 family have the enzymatic activity [19], [22], [23]. In order to isolate putative GTs responsible for the synthesis of AG polysaccharide during cotton fiber development, we searched the public cotton EST databases. One cDNA encoding a putative β-1,3- galactosyltransferase (GalT) was identified and designated *GhGalT1* (Genbank accession number: JX448620). It contains a 1053 bp of open reading frame (ORF) encoding a protein of 350 amino acids. GhGalT1 protein, which belongs to GT31 family in CAZY (http://www.cazy.org/fam/acc GT.html), contains a putative N-terminal transmembrane domain and a C-terminal conservative galactosyltransferase (GalT) functional domain (Pfam domain 01762) (Figure 1A).

A BLAST search revealed that the deduced GhGalT1 amino acid sequence has significant sequence similarity with other related galactosyltransferases (GalTs) of plants. As shown in Figure 1B, GhGalT1 shares 77%, 74% and 73% amino acid identities with *Populus* PtGalT1(XP002330737), *Arabidopsis* AtbGalT1(At1g53290) and AtbGalT2(At3g14960), respectively. Analysis of phylogenetic relationship between GhGalT1 and other GalT proteins previously reported in plants is shown in Figure 1C. This tree clearly indicated that the sixteen proteins are divided into three subgroups. GhGalT1 and PtGalT1 are located at a branch in the subgroup 1, suggesting that they are more closely related. In addition, AtbGalT1 and AtbGalT2 are also subgrouped with GhGalT1, indicating that both proteins show a close relationship with GhGalT1.

### GhGalT1 Expression in Different Cotton Tissues

**Figure 2:**
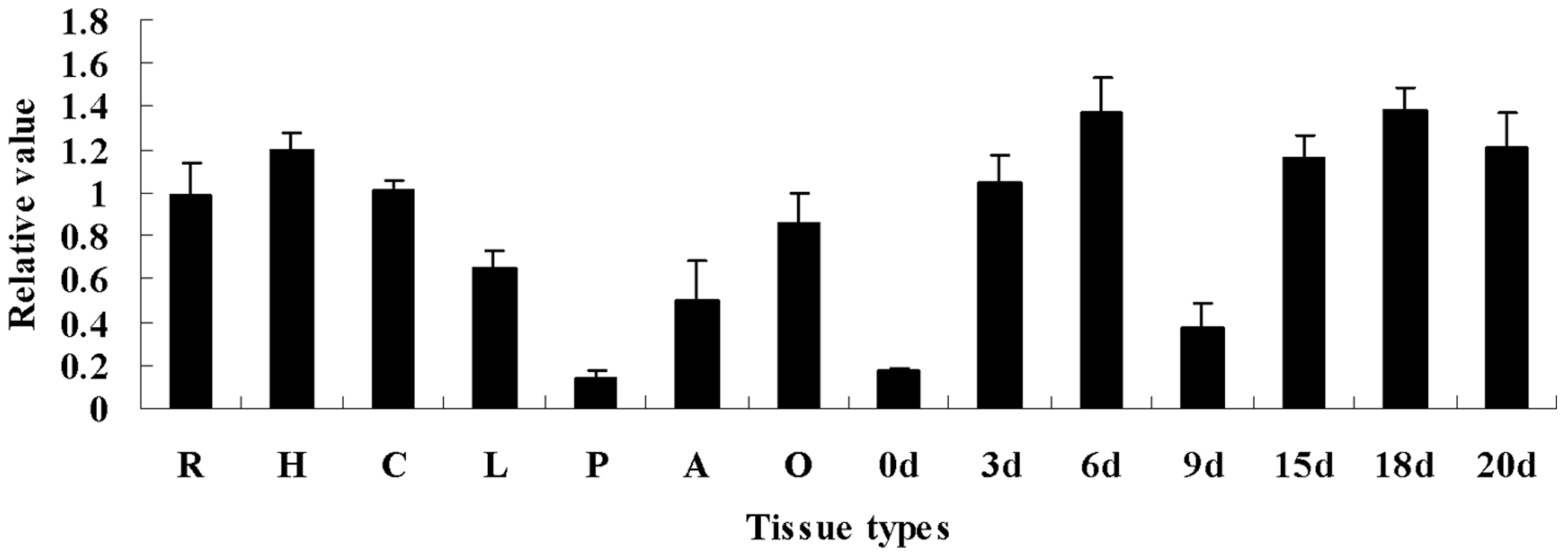
RT-PCR analysis of expression of *GhGalT1* in cotton tissues. Total RNA was isolated from different cotton tissues. R: roots; H: hypocotyls; C: cotyledons; L: leaves; P: petals; A: anthers; O: 15 DPA (day post anthesis) ovule; 0d: 0 DPA ovules and fibers; 3d: 3 DPA ovules and fibers; 6d: 6 DPA ovules and fibers; 9d: 9 DPA fibers; 15d: 15 DPA fibers; 18d: 18 DPA fibers; 20d: 20 DPA fibers.

To investigate the *GhGalT1* gene expression pattern, quantitative RT-PCR technique was employed. Total RNA was isolated from different cotton tissues and fibers at different developmental stages. The experimental results showed that *GhGalT1* was strongly expressed in hypocotyls, roots, cotyledons, ovules and fibers. During fiber development, *GhGalT1* was expressed at different levels in 0–20 DPA (days post anthesis) fibers. At early developmental stage (0–3 DPA), weak to moderate transcription of *GhGalT1* was detected in ovules and fibers. As fibers further developed, *GhGalT1* expression was gradually enhanced, and reached its peak in both 6 and 18 DPA fibers (Figure 2).

### Subcellular Localization of GhGalT1 Protein

**Figure 3:**
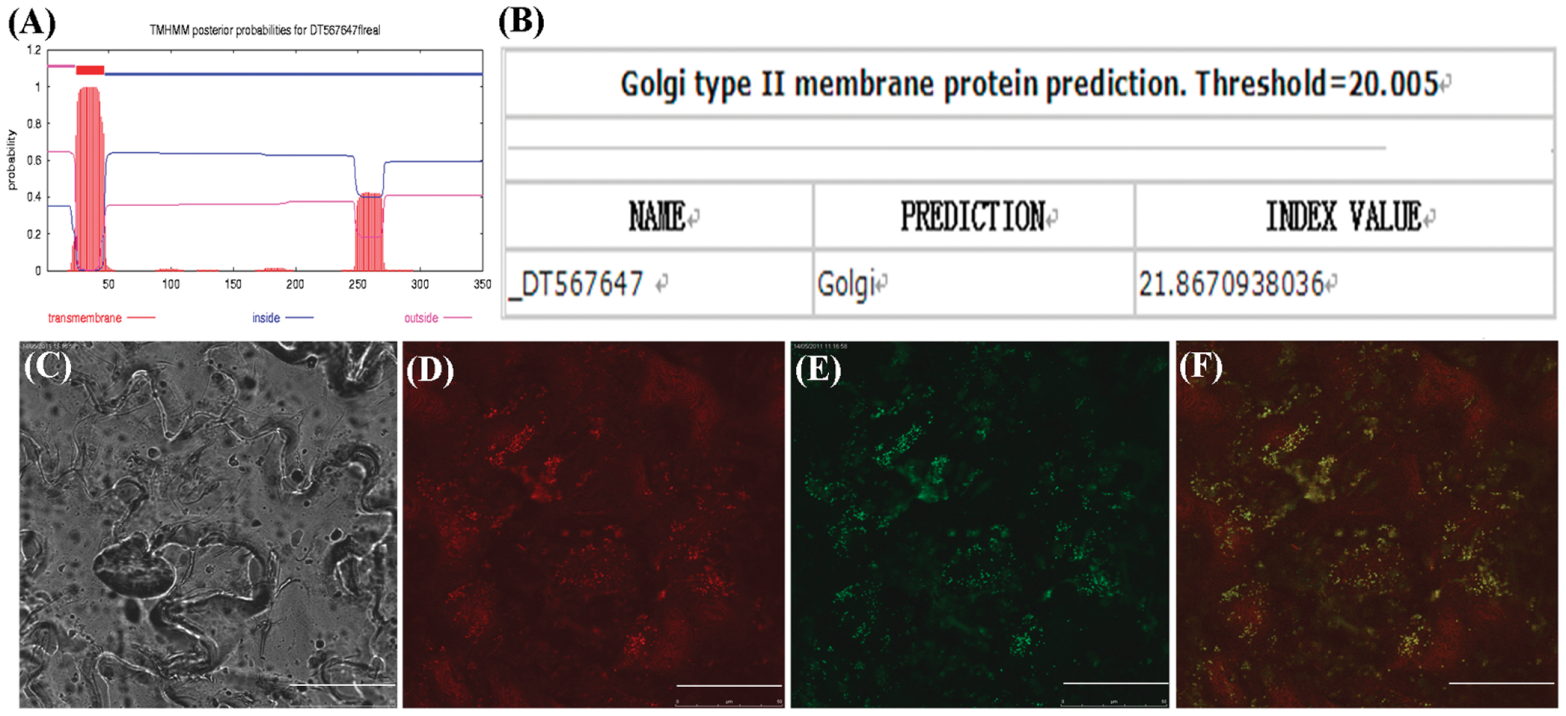
Subcellular localization of GhGalT1 protein. (**A**) The prediction of trans-membrane helices in GhGalT1 protein. GhGalT1 belongs to type II membrane proteins with an obvious single trans-membrane helice as predicted by the TMHMM2.0 program. (**B**) GhGalT1 protein with index values greater than the threshold is predicted as Golgi protein. (**C**) to (**F**) Fluorescent protein-tagged GhGalT1 fusion proteins were coexpressed with GONST1-YFP (Golgi apparatus marker) into the abaxial side of a young tobacco (*Nicotiana tabacum*) leaf epidermis. The signals were visualized with a laser confocal microscope. (**C**) Bright field photograph of leaf epidermal cells of tobacco (*Nicotiana tabacum*). (**D**) The image of GONST1-YFP (Golgi apparatus marker) fluorescence. (**E**) The image of GhGalT1-GFP fluorescence. (**F**) Image (**E**) was merged with image (**D**). Scale bar = 50 µm.

Sequence analysis using the TMHMM2.0 program (http://www.cbs.dtu.dk/service/TMHMM-2.0/) predicted that GhGalT1 belongs to the type II membrane protein with a single transmembrane helice (Figure 3A). Golgi predictor program (http://ccb.imb.uq.edu.au/golgi/golgi_predictor.shtml) predicted that GhGalT1 protein belongs to Golgi type II membrane protein (Figure 3B). To study its actual subcellular location, green fluorescent protein (eGFP)-tagged GhGalT1 protein was coexpressed with yellow fluorescent protein (YFP)-tagged GONST1, which is known to be localized in the Golgi apparatus [33], in leaf epidermis cells of tobacco (*Nicotiana tabacum*). Examination of the fluorescent signals revealed that GhGalT1 exhibited a punctate distribution, a pattern matched with GONST1 demonstrating that GhGalT1 is localized in the Golgi apparatus (Figure 3C to 3F).

### Overexpression of GhGalT1 in *Arabidopsis* Promotes Seedling Growth

**Figure 4:**
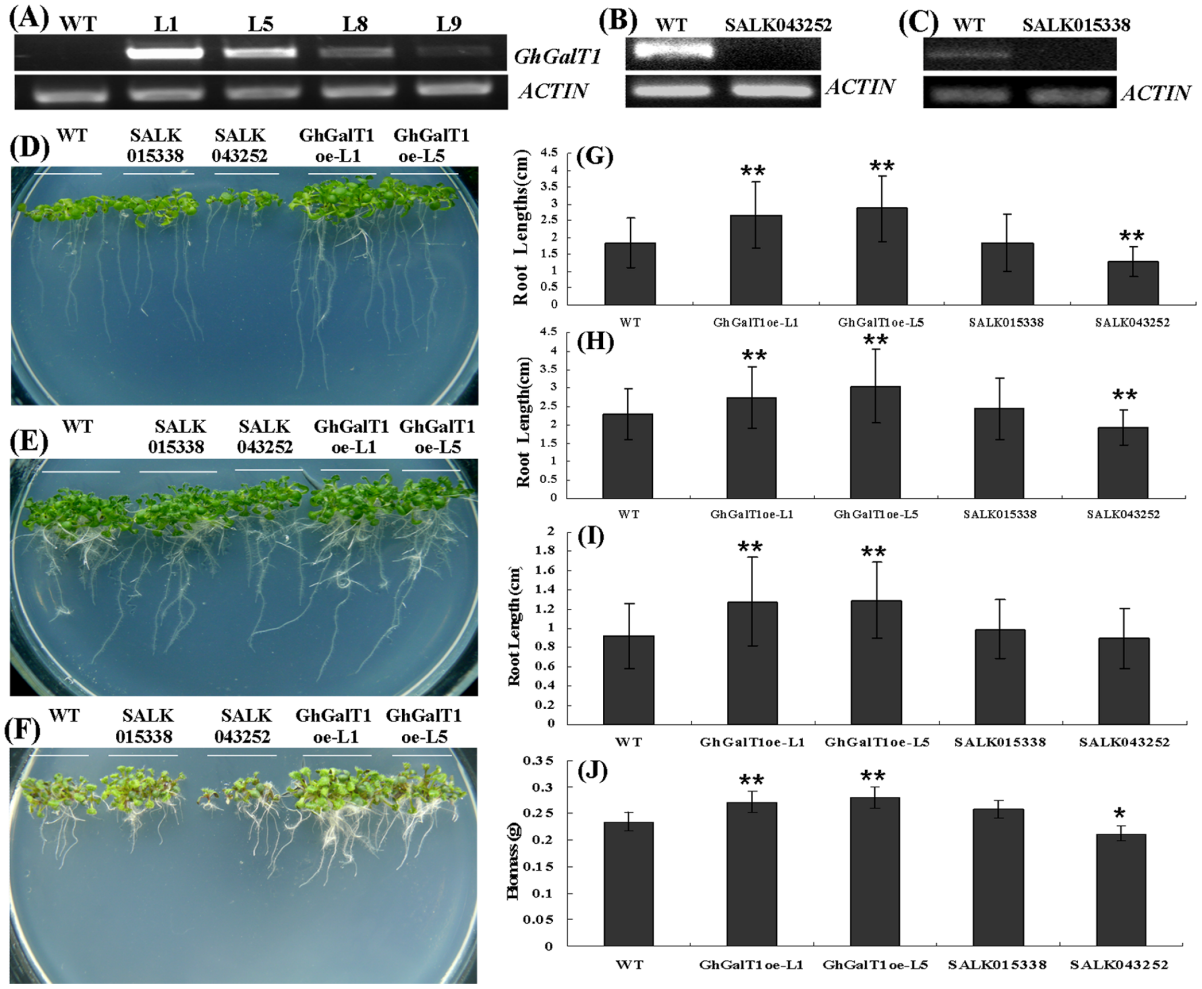
Assays in the growth status of *GhGalT1* overexpression transgenic *Arabidopsis* seedlings under light condition. (**A**) RT-PCR analysis of *GhGalT1* expression in transgenic *Arabidopsis*. (**B**) RT-PCR analysis of At3g14960 expression in SALK 043252 mutant. (**C**) RT-PCR analysis of At1g53290 expression in SALK 015338 mutant. (**D)** to (**F)** Nine-day-old seedlings of *GhGalT1* transgenic lines, wild type and mutants growing on 1/2 MS without sugar (**D**), 1/2 MS with 1% sucrose (**E**) and 1/2 MS with 100 mM sucrose (**F**). (**G)** to (**I**) Statistical analysis of the relative root length of nine-day-old seedlings of *GhGalT1* transgenic lines, wild type and mutants growing on 1/2 MS without sugar (**G**), 1/2 MS with 1% sucrose (**H**) and 1/2 MS with 100 mM sucrose (**I**). (**J**) Statistical analysis of the relative biomass (g/30 seedlings) of nine-day-old seedlings of *GhGalT1* transgenic lines, wild type and mutants growing on 1/2 MS with 1% sucrose. Error bars represent standard errors of three replicates. Asterisk represents significant difference (one asterisk: P value<0.05; two asterisk: P value<0.01) between the transgenic lines and wild-type by t-test. WT: wild type; GhGalT1oe-L1 and -L5: *GhGalT1* overexpression transgenic line 1 and 5; SALK 043252 and SALK 015338: two T-DNA insertion mutants. The assays were repeated three times along with three independent repetitions of the biological experiments.

To evaluate the role of *GhGalT1* in plant development, *GhGalT1* ORF was inserted into the plant expression vector pBI121 under the control of *CaMV35S* promoter and introduced into *Arabidopsis*. RT-PCR analysis showed that *GhGalT1* transcripts were accumulated at high levels in the nine-day-old seedlings of four homozygous *GhGalT1* transgenic lines, compared with wild type (Figure 4A). Line 1 (L1) and Line 5 (L5) which displayed high expression of *GhGalT1* were chosen for further phenotypic analysis. We also purchased two T-DNA mutants (SALK015338 and SALK043252) of two *Arabidopsis* genes (At1g53290 and At3g14960, designated as AtbGalT1 and AtbGalT2 respectively in this paper) which share high sequence similarity with GhGalT1. RT-PCR analysis demonstrated that the transcript levels of the respective genes (At1g53290 and At3g14960) were dramatically decreased in both T-DNA insertion mutants (Figure 4B and 4C).

**Figure 5:**
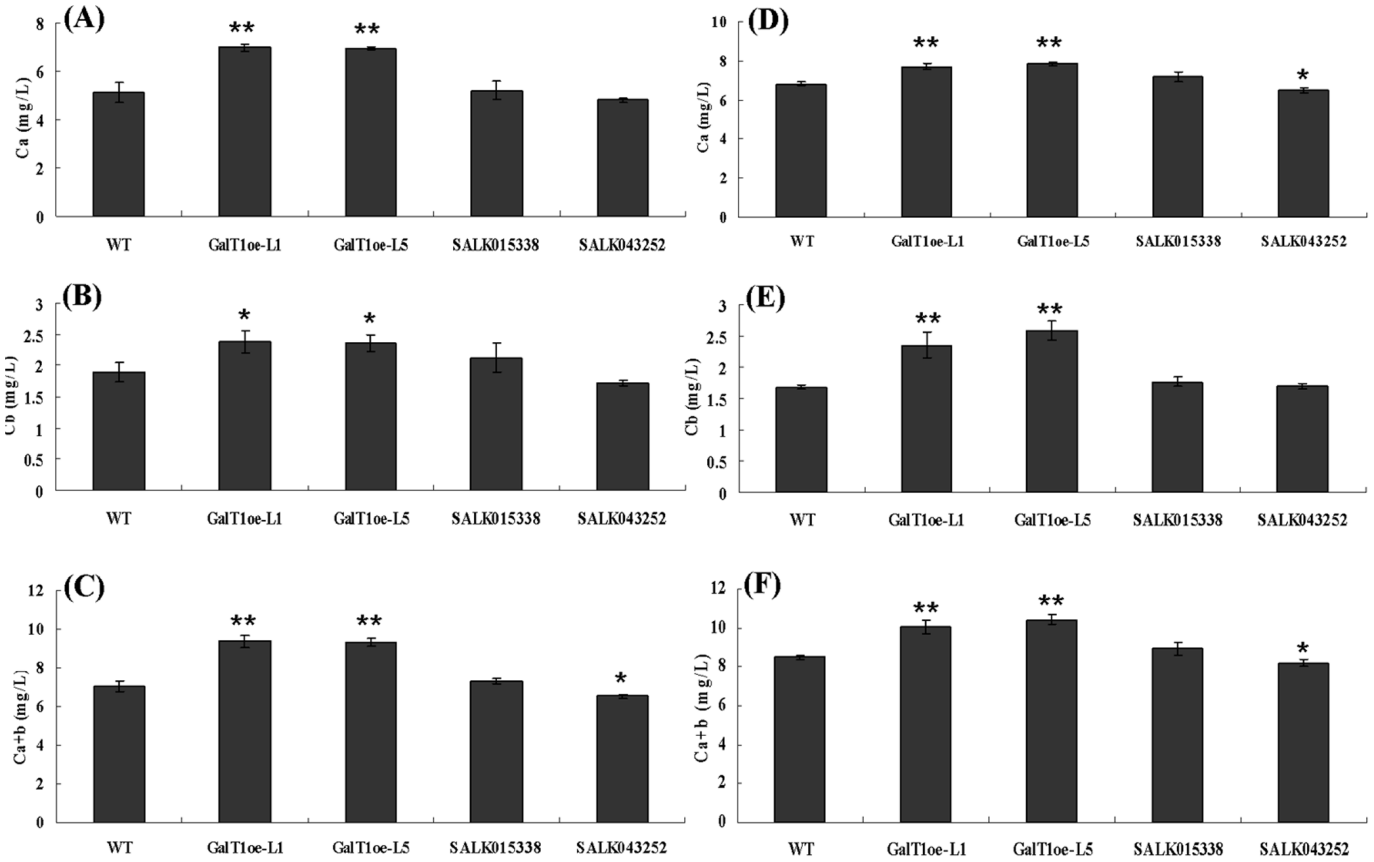
Assay of chlorophyll contents in leaves of *GhGalT1* overexpression transgenic *Arabidopsis* seedlings. Nine-day-old *Arabidopsis* seedlings of *GhGalT1* transgenic lines, wild type and mutants growing on 1/2 MS without sugar (**A–C**) and with 1% sucrose (**D–F**); Ca: chlorophyll a (mg/L); Cb: chlorophyll b (mg/L); Ca+b: total chlorophyll (mg/L); Ca = 12.7×A_663_–2.59×A_645_; Cb = 22.9×A_645_–4.67×A_663_; Ca+b = 20.3×A_645_+8.04×A_663_. A_663_ and A_645_ indicate absorbance at 645 and 663 nm. Bars show standard errors. Asterisk represents significant difference (one asterisk: P value<0.05; two asterisk: P value<0.01) between the transgenic lines and wild-type by t-test. WT: wild type; GhGalT1oe-L1 and -L5: *GhGalT1* overexpression transgenic line 1 and 5; SALK 043252 and SALK 015338: two T-DNA insertion mutants. The assays were repeated three times along with three independent repetitions of the biological experiments.

**Figure 6:**
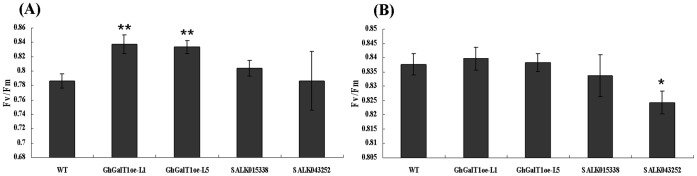
Assay of photosynthetic efficiency in leaf chlorophyll of *GhGalT1* overexpression transgenic *Arabidopsis* seedlings. Nine-day-old *Arabidopsis* seedlings of *GhGalT1* transgenic lines, wild type and mutants growing on 1/2 MS without sugar (**A**) and with 1% sucrose (**B**). Fv/Fm: the maximal of photosynthetic efficiency. Error bars represent standard errors of three replicates. Asterisk represents significant difference (one asterisk: P value<0.05; two asterisk: P value<0.01) between the transgenic lines/mutant and wild-type by t-test. WT: wild type; GhGalT1oe-L1 and -L5: *GhGalT1* overexpression transgenic line 1 and 5; SALK043252 and SALK015338: two T-DNA insertion mutants.

**Figure 7:**
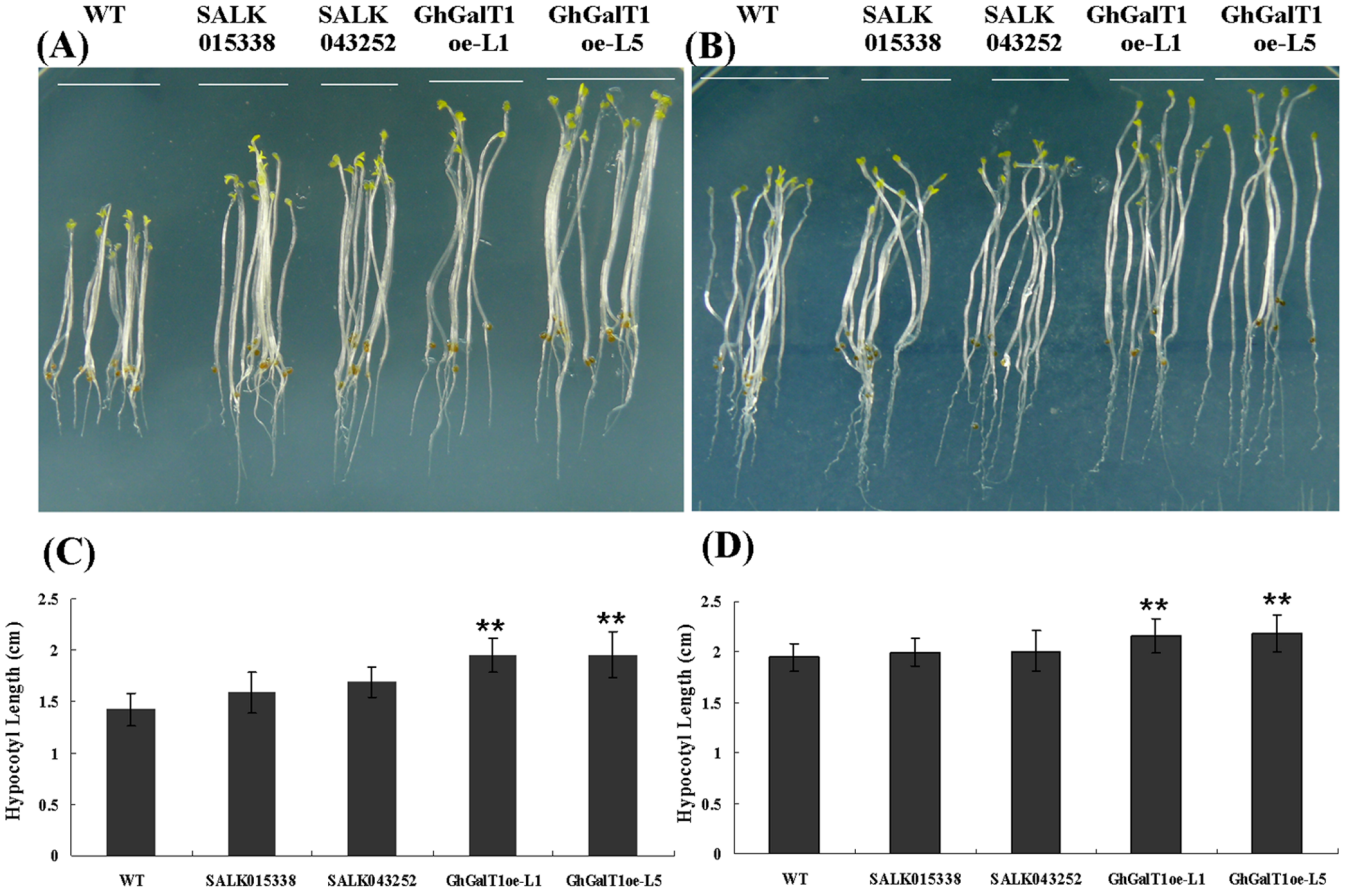
Assay in the growth status of *GhGalT1* overexpression transgenic *Arabidopsis* seedlings at early development stage under dark condition. (**A**, **B**) Nine-day-old *Arabidopsis* seedlings of *GhGalT1* transgenic lines, wild type and mutants growing on 1/2 MS without sugar (**A**) and with 1% sucrose (**B**). (**C**) Statistical analysis of the relative hypocotyl length without sugar. (**D**) Statistical analysis of the relative hypocotyl length with 1% sucrose. Error bars represent standard errors of three replicates. Asterisk represents significant difference (one asterisk: P value<0.05; two asterisk: P value<0.01) between the transgenic lines/mutant and wild-type by t-test. WT: wild type; GhGalT1oe-L1 and -L5: *GhGalT1* overexpression transgenic line 1 and 5; SALK 043252 and SALK 015338: two T-DNA insert mutants. The assays were repeated three times along with three independent repetitions of the biological experiments.

To investigate the function of *GhGalT1* gene, seeds of *GhGalT1* overexpression transgenic *Arabidopsis* (L1 and L5), SALK015338 and SALK043252 single mutants were sowed on 1/2 MS agar mediums with sucrose or without any carbon source respectively. In seed germination, wild type and *GhGalT1* transgenic plants showed no obvious difference. In seedlings growth, however, the transgenic lines grew better (Figure 4E) and have much more increased biomass than those of wild type plants (Figure 4J) under normal growth conditions (1/2MS with 1% sucrose). At any given time, the root length in wild type plants grown on medium supplemented with different concentrations of sucrose is remarkably shorter than that of *GhGalT1* transgenic plants, but longer than SALK 043252 single mutant plants (Figure 4D to 4F). Especially on the medium without any carbon source, the transgenic seedlings have much longer primary roots (Figure 4D), higher chlorophyll content (Figure 5) and higher photosynthetic efficiency (Figure 6), compared with wild type and mutants. There was a significant difference in root length between the transgenic lines and wild type in the medium with different concentrations of sucrose (Figure 4G to 4I). Furthermore, under dark conditions, the transgenic seedlings growing on 1/2 MS medium with 1% sucrose or without any carbon source have much longer hypocotyls than those of wild type (Figure 7). These results suggested that *GhGalT1* may be involved in regulating carbohydrate metabolism required for seedling growth and development.

### Overexpression of *GhGalT1* in *Arabidopsis* Enhances Plant Tolerance to Exogenous D-arabinose and D-galactose

**Figure 8:**
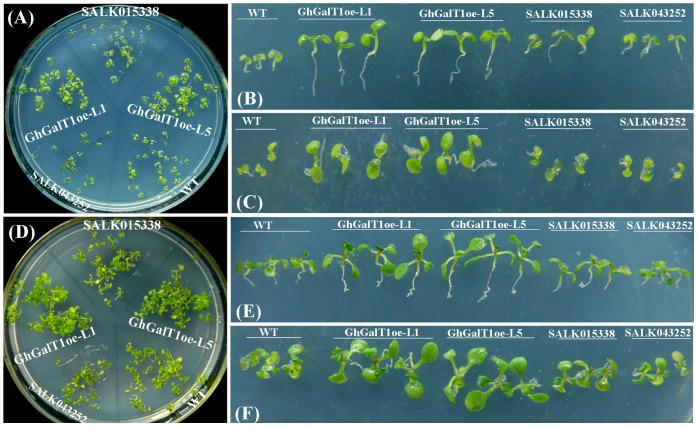
Overexpression of *GhGalT1* gene in *Arabidopsis* enhanced plant tolerance to exogenous D-arabinose and D-galactose. Nine-day-old *Arabidopsis* seedlings of *GhGalT1* transgenic lines, wild type and mutants growing on 1/2 MS with 75 mM D-arabinose (**A**–**C**) and 75 mM D-galactose (D–F), respectively. WT: wild type; GhGalT1oe-L1 and -L5: *GhGalT1* overexpression transgenic line 1 and 5; SALK 043252 and SALK 015338: two T-DNA insertion mutants. The assays were repeated three times along with three independent repetitions of the biological experiments.

Under exogenous D-galactose and D-arabinose treatments, seedlings of wild type, SALK043252 and SALK015338 mutants showed severe growth defects, such as increasingly stunted roots and more variable root hairs in length, and inhibited shoot growth. In contrast, *GhGalT1* transgenic seedlings were much less sensitive to exogenous D-arabinose (Figure 8A to 8C) and D-galactose (Figure 8D to 8E). *GhGalT1* transgenic seedlings growing on 1/2MS medium with 75 mM D-arabinose or D-galactose displayed much bigger cotyledons and rosette leaves, and longer roots, compared with those of wild type (Figure 8). These data suggested that overexpression of *GhGalT1* in *Arabidopsis* enhanced tolerance to D-arabinose and D-galactose.

### Overexpression of *GhGalT1* in *Arabidopsis* Changes Cell Wall Pectin of Transgenic Plants

**Figure 9:**
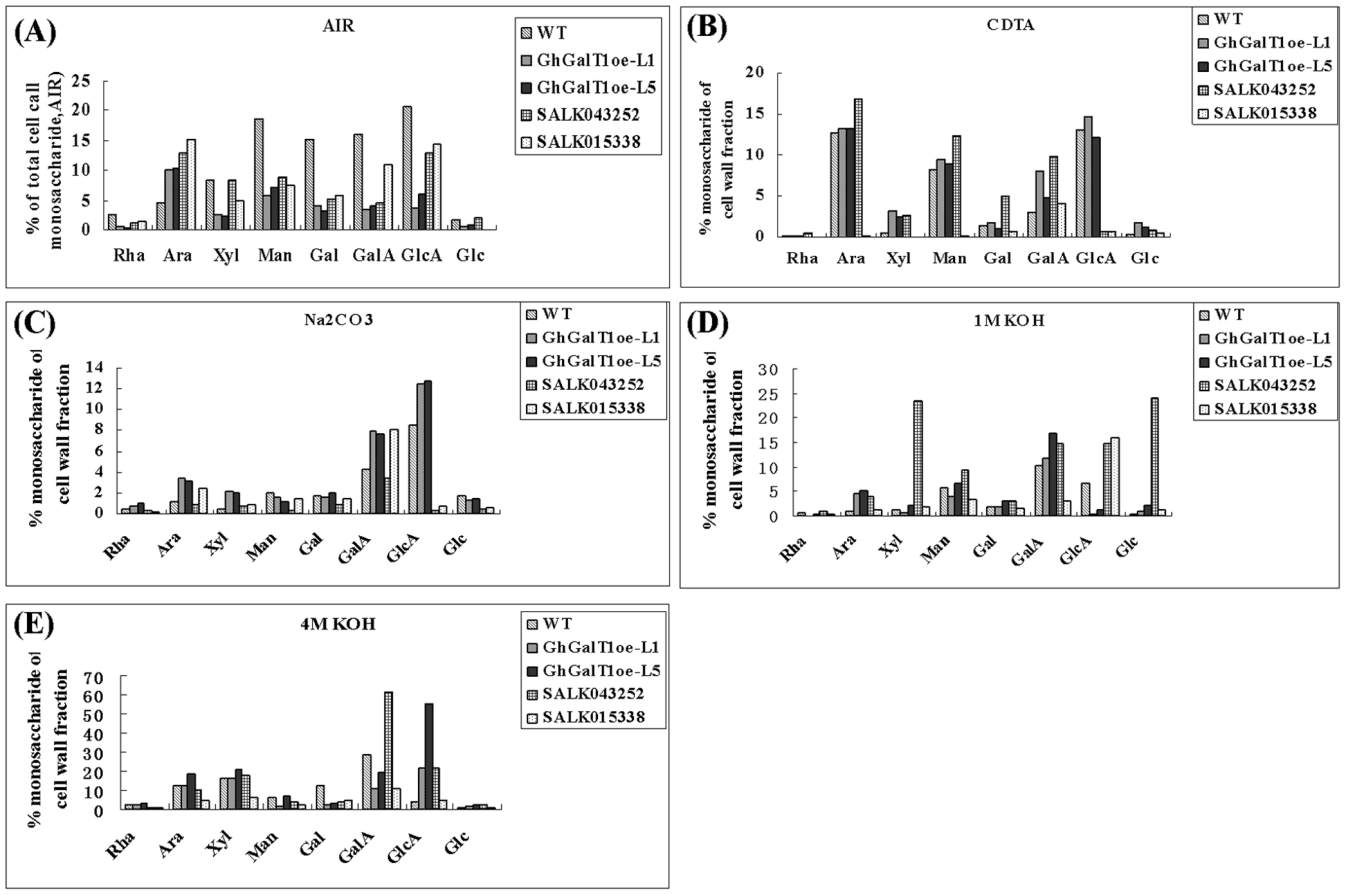
Analysis of cell wall polymer fractions in the *GhGalT1* overexpression transgenic *Arabidopsis* plants. (**A**) Non-cellulosic monosaccharide composition of alcohol insoluble residue (AIR) of rosette leaves from *GhGalT1* transgenic lines, wild type and mutants. (**B**–**E**) Sequential extraction of the extracted alcohol-insoluble residue (AIR) and analysis of monosaccharide composition. AIR of rosette leaves from *GhGalT1* transgenic lines, wild type and mutants was sequentially extracted using CDTA (**B**), Na_2_CO_3_ (**C**), 1 M KOH (**D**), and 4 M KOH (**E**). Arabinose (Ara), rhamnose (Rha), xylose (Xyl), mannose (Man), galactose (Gal), glucose (Glc), galacturonic acid (GalA) and glucurnic acid (GlcA) are expressed as a percentage of the respective fractions. WT: wild type; GhGalT1oe-L1 and -L5: *GhGalT1* overexpression transgenic line 1 and 5; SALK 043252 and SALK 015338: two T-DNA insertion mutants.

In order to determine which specific cell wall polysaccharides are affected by the overexpression of *GhGalT1*, cell wall alcohol-insoluble residue (AIR) was prepared from the rosette leaves of wild type, the *GhGalT1* overexpression transgenic lines (L1 and L5), SALK015338 and SALK043252 single mutants. Chemical fractionation was also employed to separate the various cell-wall polysaccharides. The monosaccharide contents of pectin-rich (trans-diamino-cyclohexane-N, N, N, N, tetraacetic acid (CDTA), Na_2_CO_3_) and hemicellulose-rich (1 M KOH, 4 M KOH) fractions were analyzed by gas chromatography (GC). As shown in Figure 9, there was a remarkable increase in arabinose (Ara), xylose (Xyl), galacturonic acid (GalA) in *GhGalT1* transgenic lines (L1 and L5), compared with that of wild type and mutants, in Na_2_CO_3_ fraction, suggesting that the pectin was changed in transgenic cell walls.

**Figure 10:**
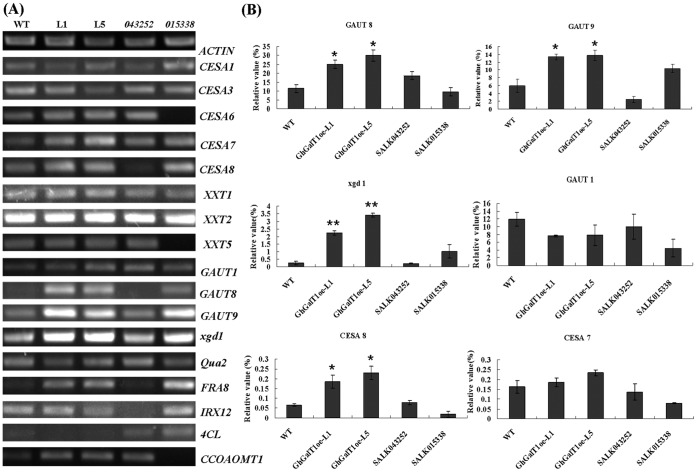
RT-PCR analysis of expression of the cell wall-associated genes in the *GhGalT1* overexpression transgenic *Arabidopsis* plants. The expression levels of genes involved in the biosynthesis of cellulose in primary cell wall (*CesA1*, *CesA3* and *CesA6*), cellulose in secondary cell wall (*CesA4*, *CesA7* and *CesA8*), xyloglucan (*XXT1*, *XXT2* and *XXT5*), xylan (*FRA8* and *IRX12*), pectin (*GAUT1*, *GAUT8*, *GAUT9*, *Qua2* and *xgd1*) and lignin (*4CL* and *CCoMoAT1*) were examined in two-week-old seedlings of *GhGalT1* transgenic lines, wild type and mutants, using *ACTIN2* as an internal control. (**A**) Semi-quantitative RT-PCR analysis. (**B**) Quantitative RT-PCR analysis. Error bars represent standard errors of three replicates. Asterisk represents (very) significant difference (one asterisk: P value<0.05; two asterisk: P value<0.01) between the transgenic lines/mutant and wild-type by t-test. WT: wild type; GhGalT1oe-L1 and -L5: *GhGalT1* overexpression transgenic line 1 and 5; SALK043252 and SALK015338: two T-DNA insert mutants. The assays were repeated three times along with three independent repetitions of the biological experiments.

Furthermore, expression of the genes related to the synthesis of different cell wall fractions was examined in the transgenic plants. As shown in Figure 10, RT-PCR analysis revealed that expression of three genes involved in pectin biosynthesis (*GAUT8*, *GAUT9* and *xgd1*) was up-regulated dramatically in the transgenic lines, suggesting that overexpression of *GhGalT1* in *Arabidopsis* may affect the pectin biosynthesis. In addition, the expression of *CesA8* gene involved in the biosynthesis of cellulose in secondary cell wall was up-regulated in the transgenic lines. On the contrary, the expression levels of other genes involved in the biosynthesis of cellulose (*CesA1*, *CesA3*, *CesA6*, *CesA4* and *CesA7*), xyloglucan (*XXT1*, *XXT2* and *XXT5*), xylan (*FRA8* and *IRX12*), pectin (*Qua2* and *xgd1*) and lignin (*4CL* and *CCoMoAT1*) showed no obvious changes in the transgenic lines, compared with that of wild type. These data suggested that *GhGalT1* may be involved in regulation of pectin biosynthesis required for plant development.

## Discussion

AGPs are generally regarded as one of the most complex families of macromolecules in plants. They are characterized by the extensive O-glycosylation of the protein backbone which occurs post-translationally in the Golgi apparatus [4]. Typically, the glycan side chains account for more than 90% w/w of the mass of the glycoprotein and consist predominantly of arabinose and galactose residues [3]. Much progress on the functions of AGPs has been made by using AGP T-DNA mutants. However, precise roles of AGP glycosylation remain elusive and little is known about specific glycosyltransferases involved in the biosynthesis of AG-glycans. Strasser et al (2007) searched the *Arabidopsis* genomic databases using mammalian β-(1,3)-GalTs and identified one homolog, GALT1 (At1g26810). Functional analysis revealed that GALT1 possesses β-(1,3)-GalT activity involved in the formation of the Lewis A structure on *N*-glycans in *Arabidopsis thaliana* [20]. Qu et al. (2008) identified and systematically characterized 20 putative GalTs responsible for synthesizing the β-(1,3)-Gal linkage in *Arabidopsis thaliana* by a similar approach [19]. On the basis of these studies, AGs on AGPs are hypothesized to be synthesized by type II ER/Golgi-targeted GTs, including members of GT31 family that have β-(1,3) GalT activity. The principal β-(1,3)-Gal-containing molecules in plants are the arabino-(3,6)-galactan (AG) chains on AGPs [39]. Recently, Egelund et al. (2011) classified all the putative GalTs (including ninety-four sequences from plants) into CAZy GT31 family, which are phylogenetically identified to 11 distinct clades. Plant specific β-(1,3)-GalT-containing clades 7 and 10 are speculated to be primarily involved in the assembly of the glycan moiety of AGPs [24]. In this study, we identified a putative β-1,3-galactosyltransferase (GhGalT1) in cotton. GhGalT1 is closely related with At3g14960 and At1g53290 in plant specific clade 10, suggesting it might be involved in AGP biosynthesis. Because generating transgenic cotton is time-consuming and labor-intensive, we firstly expressed this gene in *Arabidopsis* and the intriguing results showed that GhGalT1 might be involved in the regulation of pectin synthesis.

A recent study revealed that AtFUT4 and AtFUT6 of GT37 family could add fucose to the end of AG side chain on AGP. They are the first enzymes to be characterized that are specific for AGP glycosylation. Also, AtFUT6 was localized to the Golgi apparatus [21]. In this study, sequence analysis indicated that GhGalT1 is type II membrane protein that contains a short cytoplasmic N-terminus followed by a single transmembrane helix and a long non-cytoplasmic C-terminus. Subcellular localization analysis demonstrated that GhGalT1 is targeted to the Golgi apparatus, in agreement with the hypothesis that AGP glycosylation takes place in the Golgi apparatus or endoplasmic reticulum (ER) [33], [40].

Characterizing AGP-specific GT mutants is a good approach to address the functional importance of the glycan moiety of AGPs [6]. However, we did not observe significant phenotypic changes of the single T-DNA insertion mutant of both At3g14960 and At1g53290 in inflorescence height, rosette leave size, flowering time, etc. It has been reported that At3g14960, At1g53290 and At2g26100 are closely subgrouped together in plant specific clade 10 [24]. It is possible that these genes may perform redundantly biochemical functions in *Arabidopsis*. Functional redundancy have been reported for many glycosyltransferases, such as GTs (IRX9 and IRX9L, IRX14 and IRX14L, FRA8 and F8H) involved in xylan synthesis, GTs (XXT1 and XXT2) involved in xyloglucan synthesis [40], [41], [42]. Therefore, double and triple mutants are needed for the phenotypic analysis.

Previous reports have documented deleterious physiological effects in the presence of excess galactose, such as abnormal root elongation, root tip necrosis, leaf chlorosis, growth arrest [43]. More recently, Egert et al (2012) found that an *Arabidopsis* galactokinase mutant (AtGALK, At3g06580) can target free Gal to the vacuole and is insensitive to exogenous galactose [44]. In our study, overexpression of *GhGalT1* in *Arabidopsis* enhanced tolerance to galactose and arabinose, resulting in bigger cotyledons and rosette leaves, longer roots in the transgenic plants. Given the data together, we speculated that GhGalT1 may be responsible for transferring galactose and/or arabinose to AG polysaccharide of AGPs, thus detoxification of exogenous galactose and/or arabinose.

Dicot plant cells are composed of type I cell walls, which are characterized by a cellulose-xyloglucan framework with approximately equal amounts of cellulose microfibrils and xyloglucans. The cellulose-xyloglucan framework is typically embedded in a network of ample pectic polysaccharides, which mainly consist of homogalacturonans (HGA), rhamnogalacturonan I (RG-I) and rhamnogalacturonan II (RGII) [1], [45]. AGPs are very likely to be one form of covalent cross linker for wall matrix phase polysaccharides. Several lines of evidence have shown that AGPs/AGs could interact with pectin [6]. It was proposed that Rha residues on AG side chains of AGPs might be the attachment sites for RG-I [46]. Therefore, Alteration of sugars on AG side chains of AGPs may change the interactive molecular surface and affect the interaction of AGPs and pectin. In our study, RT-PCR results showed that expression of three genes involved in pectin biosynthesis (*GAUT8*, *GAUT9* and *xgd1*) were up-regulated in the transgenic plants. Further analysis of monosaccharide composition of cell wall fractions also showed that pectin was changed in transgenic plants. Since no evidence have shown that plant galactosyltransferases from CAZy GT31 family are implicated in the synthesis of pectin, our results suggested that GhGalT1 may be involved in the synthesis of glycan moieties of AGPs and then affects synthesis of pectin in cell walls. However, the definite function of GhGalT1 in AGP as well as pectin synthesis in cotton development still need to be dissected in detail. Further biochemical characterization of GhGalT1 will provide insights to unravel the mechanism and pathway of AGP synthesis.
